# Renal Podocyte Injury in a Rat Model of Type 2 Diabetes Is Prevented by Metformin

**DOI:** 10.1155/2012/210821

**Published:** 2012-09-27

**Authors:** Junghyun Kim, Eunjin Shon, Chan-Sik Kim, Jin Sook Kim

**Affiliations:** Traditional Korean Medicine (TKM) Based Herbal Drug Research Group, Herbal Medicine Research Division, Korea Institute of Oriental Medicine, 1672 Yuseongdaero, Yuseong-gu, Daejeon 305-811, Republic of Korea

## Abstract

Hyperglycemia promotes oxidative stress and hence generation of reactive oxygen species (ROS), which is known to play a crucial role in the pathogenesis of diabetic nephropathy. Metformin, an oral hypoglycemic drug, possesses antioxidant effects. The aim of this paper is to investigate the protective effects of metformin on the injury of renal podocytes in spontaneously diabetic Torii (SDT) rats, a new model for nonobese type 2 diabetes. Metformin (350 mg/kg/day) was given to SDT rats for 17 weeks. Blood glucose, glycated haemoglobin (HbA1c), and albuminuria were examined. Kidney histopathology, renal 8-hydroxydeoxyguanosine (8-OHdG) levels and apoptosis were examined. In 43-week-old SDT rats, severe hyperglycemia was developed, and albuminuria was markedly increased. Diabetes induced significant alterations in renal glomerular structure. In addition, urinary and renal 8-OHdG levels were highly increased, and podocyte loss was shown through application of the TUNEL and synaptopodin staining. However, treatment of SDT rats with metformin restored all these renal changes. Our data suggested that diabetes-induced podocyte loss in diabetic nephropathy could be suppressed by the antidiabetes drug, metformin, through the repression of oxidative injury.

## 1. Introduction

Diabetic nephropathy is one of the most serious complications of both type 1 and type 2 diabetis mellitus [1]. The clinical hallmarks of diabetic nephropathy include progressive albuminuria followed by a gradual decline in renal function [2]. The loss of glomerular podocytes precedes and predicts the onset of clinical nephropathy and may be an early pathological manifestation of diabetic nephropathy [3, 4]. Among the three intrinsic cells in glomerulus, podocyte is one of the important ingredients of filtration barrier which has special cytobiological trait and physiological function. The injury of podocyte can inevitablely lead to the occurrence of proteinuria [5]. Patients with advanced diabetic nephropathy have few treatment options and often have a poor prognosis [6].

Metformin has been widely used for treating type 2 diabetes without the stimulation of insulin production [7]. In previous study, metformin could reduce macrovascular morbidity and mortality, suggesting that it achieved its antiatherogenic, anti-inflammatory, and antioxidant effects [8, 9]. Moreover, metformin significantly decreased the urine albumin excretion rate in patients with type 2 diabetes [10]. The benefits of metformin with regard to the risk of cardiovascular outcomes and metabolic parameters suggest its clinical use in treating chronic kidney disease [11]. The precise mechanisms beyond the effect of metformin on glucose still are obscure. Recent studies suggest that therapeutic effect of metformin might be mediated by its action on AMP-activated kinase (AMPK) in peripheral tissues [12, 13]. Other studies show that metformin decreases intracellular ROS [14, 15].

Diabetic animal models have a critical role in the elucidation of the mechanisms of diabetic complications and the development of novel drugs as treatments. The spontaneously diabetic Torii (SDT) rat is a new model for nonobese type 2 diabetes that spontaneously develops hyperglycemia and glucose intolerance resulting from decreased insulin secretion accompanying *β*-cell degeneration [16]. SDT rat is an ideal model for investigating diabetic complications with gradual progression and is the only diabetic model known with progression to proliferative retinopathy [17, 18]. 

In the present study, we examined the utility of the SDT rat as a model of diabetic nephropathy, and investigated the preventive effect of metformin on the injury of diabetic glomerular podocytes in SDT rats.

## 2. Materials and Methods

### 2.1. Animals 

The experiments were performed according to the National Institutes of Health (NIH) Guide for the Care and Use of Laboratory Animals and approved by the Korea Institute of Oriental Medicine Institutional Animal Care and Use Committee. Male SDT rats and age-matched Sprague-Dawley (SD) rats were purchased from CLEA Japan (Tokyo, Japan) and acclimated for 1 week prior to the study. Rats were individually housed in plastic cages and maintained at 24°C ± 2°C with a 12 h light : dark cycle and received a basal diet (ground Purina rat chow, Ralston Purina, MO, USA) and tap water ad libitum. At 25 weeks of age, the animals were randomly divided into three groups as follows: (1) normal SD rats (NOR, *n* = 10), (2) vehicle-treated SDT rats (SDT, *n* = 10), and (3) SDT rats treated with metformin (SDT + MET, 350 mg/kg body weight). Metformin was administered once a day orally for 17 weeks. The blood glucose level and body weight were monitored consecutively, and glycated hemoglobin was determined by a commercial kit (Unimate HbA1c, Roche Diagnostics, Mannheim, Germany). 

### 2.2. Metabolic and Morphological Analysis

When the rats reached 43 weeks of age, blood glucose and HbA1c (A1C) were measured using an automated analyzer (Wako, Japan). Blood samples were collected from the tail vein after a 16 h fast. Individual rats were placed in metabolic cages to obtain 24 h urine collections, and daily urinary albumin excretion levels were measured. Renal cortexes were fixed in 10% formaldehyde and embedded in paraffin, and 4 *μ*m thick sections were prepared. The sections were stained with periodic acid-Schiff (PAS) reagent or Masson's modified trichrome to assess glomerulosclerosis and demonstrate collagenous tubulointerstitial matrix, respectively.

### 2.3. Measurement of Urinary 8-Hydroydeoyguanosine (8-OhdG)

24 h urine samples were collected from rats. The samples were purged of air with a steam of nitrogen to prevent the artificial formation of 8-OHdG and stored frozen at −80°C until analyzed. Urine samples were centrifuged at 2,000 g for 20 min, and after proper dilution, the supernatant was used for the determination of 8-OHdG by a competitive enzyme-linked immunosorbent assay (ELISA) kit (Japan Institute for the Control of Aging, Fukuroi, Japan). The urinary 8-OHdG was expressed as total amounts excreted in 24 h. 

### 2.4. Measurement of 8-OHdG Levels in Renal Tissues

The kidney was rapidly excised. The renal cortex tissues were homogenized in 5 mL of 50 mmol/L Tris-HCl (pH 7.4). The homogenates were centrifuged at 800 g for 10 min to precipitate nuclear fraction, and 1 mL of solution containing 10 mmol/L EDTA, 10 mmol/L Tris-HCl (pH 8.0), 150 mmol/L NaCl, and 0.2% SDS was added. After homogenization, the mixture was incubated at 56°C for 70 min with proteinase K (700 *μ*g/mL) and then heated at 95°C for 10 min. DNA was extracted with equal volumes of phenol, chloroform, and isoamyl alcohol (25 : 24 : 1) and then with chloroform. DNA was precipitated with 70% ethanol at −20°C for 2 h. DNA was resuspended in 10 mmol/L Tris-HCl and 0.1 mmol/L EDTA (pH 8.0). Five microliters of 200 mmol/L sodium acetate buffer (pH 4.8) and 5 *μ*g nuclease P1 (Invitrogen, CA, USA) were added to 45 *μ*L DNA samples. The mixtures were incubated at 37°C for 1 h to digest the DNA to nucleotides. Then, 5 *μ*L of 500 mmol/L Tris-HCl (pH 8.0), 10 mmol/L MgCl_2_, and 0.6 units alkaline phosphatase (Toyobo, Osaka, Japan) were added to the samples. The mixtures were incubated at 37°C for 1 h to hydrolyze the nucleotides to nucleosides. The nucleoside samples were used for the determination of 8-OHdG by competitive ELISA kit as described above. 

### 2.5. Double Labelling for TUNEL and Synaptopodin Expression

TUNEL was performed with the In-situ cell death detection kit AP (Roche Diagnostics, Mannheim, Germany) according to the manufacturer's instructions. Apoptotic cells were detected with a colour solution containing nitroblue tetrazolium (NBT, Roche Diagnostics) and 5-bromo-4-chloro-3-indolylphosphate (BCIP, Roche Diagnostics). Labelling with mouse anti-synaptopodin antibody (Santa Cruz Biotechnology, CA, USA) was performed subsequently on the same sections with secondary detection by horseradish peroxidase-conjugated anti-mouse IgG antibody (Santa Cruz) and an AEC Red peroxidase substrate kit (Vector Laboratories, Burlingame, California). To prevent cross-reaction between the two labelling procedures the slides were incubated with normal mouse serum (Dako) after the TUNEL labelling. For morphometric analysis, the areas of positive signal for synaptopodin per glomerulus over a total of 40 glomeruli was determined using Image J software, and cells dually labelled for TUNEL and synaptopodin were counted.

### 2.6. Immunohistochemical Staining for WT-1

 Staining was performed as previously described [19]. Antibody was rabbit anti-Wilms tumor antigen-1 (WT-1, 1 : 250, Santa Cruz). The sections were visualized by nitroblue tetrazolium chloride (NBT) and 5-bromo-4-chloro-3-indolyl phosphate (BCIP) substrate staining and counterstained with methyl green. Negative controls for immunohistochemistry were run by incubating the sections with nonimmune serum instead of the primary antibody. For morphometric analysis, the positive cell numbers per glomerulus in a total of 40 glomerulus was determined.

### 2.7. Western Blotting Analysis in Retinal Tissues

Proteins were extracted from renal tissues, and then 20 *μ*g of protein lysates was separated by SDS-polyacrylamide gel electrophoresis and transferred to nitrocellulose membranes (Biorad, CA, USA). Membrane was probed with mouse anti-AMPK antibody (Santa Cruz) and mouse anti-phospho-AMPK antibody (Santa Cruz), and then the immune complexes were visualized with an enhanced chemiluminescence detection system (Amersham Bioscience, NJ, USA).

### 2.8. Statistical Analysis

Data are expressed as mean ± SE and analyzed by one-way analysis of variance (ANOVA) followed by Tukey's multiple comparison test or by unpaired Student's *t-*test using GraphPad *Prism 5.0* software (Graph pad, San Diego, CA, USA). Differences with a value of *P* < 0.01 were considered statistically significant.

## 3. Results

### 3.1. Body Weight and Metabolic Parameters in Blood

In SDT diabetic rats at 43 weeks of age, body weight was decreased compared with normal rats and did not change compared with rats that treated metformin. Blood glucose and HbA1c levels were significantly increased in SDT rats (*P* < 0.01 versus normal rats). Metformin induced a minor decrease of blood glucose and HbA1c levels (Table 1).

### 3.2. Morphology and Renal Function

**Figure 1:**
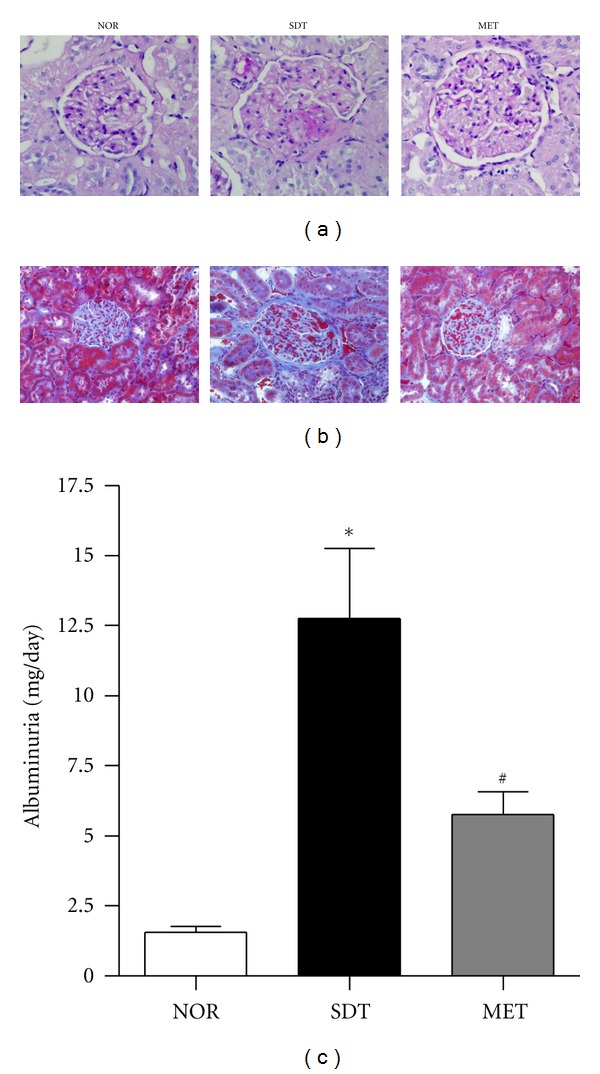
Renal histopathology and function(a). Periodic acid-Schiff staining of glomeruli, ×400 magnification (b). Masson's trichrome stained renal sections, ×400 magnification (c). Albuminuria in normal rat (NOR), spontaneously diabetic Torii rat (SDT) and SDT rat treated with 350 mg/kg metformin (MET). All data are expressed as mean ± SE (*n* = 8). *P < 0.01 versus NOR group, ^#^ *P* < 0.01 versus SDT group.

Mesangial matrix expansion is considered a hallmark of diabetic nephropathy. At 43 weeks of age, SDT rats showed focal mesangial matrix expansion, tubulointerstitial damage, and albuminuria were significantly increased in SDT rats compared to normal rats. Metformin treatment ameliorated mesangial expansion and albuminuria, compared with untreated SDT rats (Figures 1(a) to 1(c)).

### 3.3. Quantification of 8-OHdG in Urine and Renal Tissue

**Figure 2:**
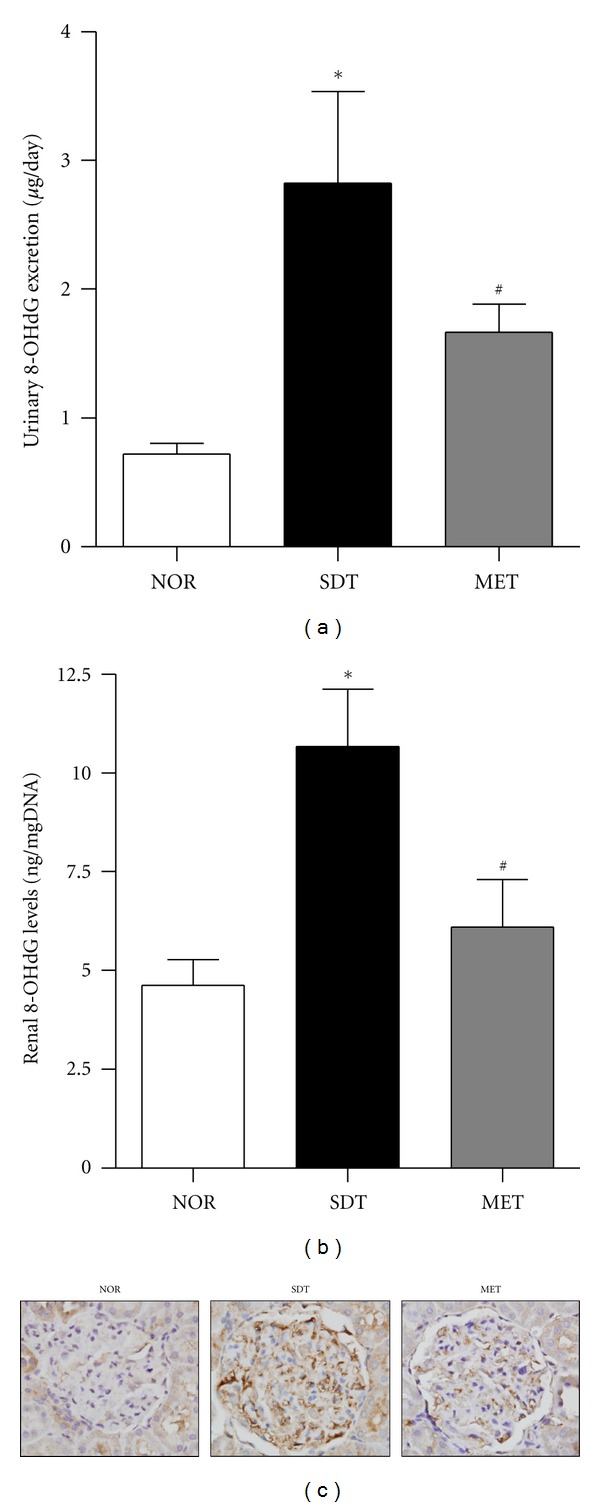
Expression of 8-OHdG in urine and renal tissues. (a) Uriany 8-OHdG excretion levels, (b) renal 8-OHdG levels, and (c) immunohistochemical stating for 8-OHdG in normal rat (NOR), spontaneously diabetic Torii rat (SDT) and SDT rat treated with 350 mg/kg metformin (MET). All data are expressed as mean ± SE (*n* = 8). *P < 0.01 versus NOR group, ^#^ *P* < 0.01, versus SDT group.

The total amounts of urinary 8-OHdG excretion were significantly greater in SDT rats than in normal rats (Figure 2(a)). The levels of 8-OHdG in renal cortex tissues were significantly increased in SDT rats as compared with those from the normal rats (Figure 2(b)). Similarly, There was increased expression of 8-OHdG in the podocytes of SDT rats and this labelling had a nuclear and/or perinuclear localization (Figure 2(c)). Metformin reduced these diabetes-induced increases in urinary and renal 8-OHdG.

### 3.4. Apoptosis of Renal Podocytes in SDT Rats

**Figure 3:**
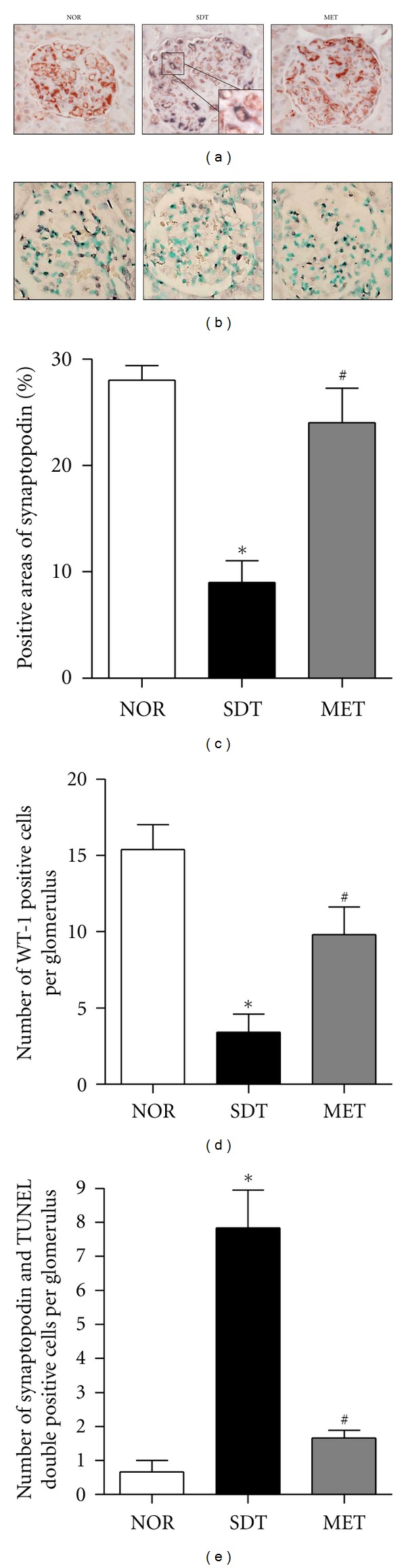
Podocyte loss. A representative photomicrograph of (a) dual labelling for TUNEL (black) and synaptopodin (red) and (b) WT-1 (black). ×400 magnification. Double-positive cells (magnified inset) indicates the colocalization of the podocyte marker and apoptosis. Quantitative analyses of (c) positive areas of synaptopodin, (d) positive cells of WT-1 and (e) TUNEL and synaptopodin-double-positive cells. All data are expressed as mean ± SE (*n* = 8). *P < 0.01 versus NOR group, ^#^ *P* < 0.01, versus SDT group.

In SDT rats, many podocytes, mesangial cells, and capillary endothelial cells were positively labelled by the TUNEL technique. However, metformin prevented the increase in the positive cells that was seen in normal kidney. Average numbers of podocytes per glomerular section were determined by counting cells and measuring areas that were positively labeled with two podocyte markers, such as synaptopodin and WT-1 In SDT rats, synaptopodin and WT-1-positive cell counts tended to decrease compared with age-matched normal rats. Treatment with metformin visibly increased the positive cells and areas in the kidney glomeruli (Figures 3(a) to 3(d)). In dual-labelled sections, TUNEL-positive cells were localized to the regions of synaptopodin expression consistent with apoptosis of podocytes in diabetic renal tissue (Figures 3(a) and 3(e)).

### 3.5. Changes in AMPK Activation in SDT Rats

**Figure 4:**
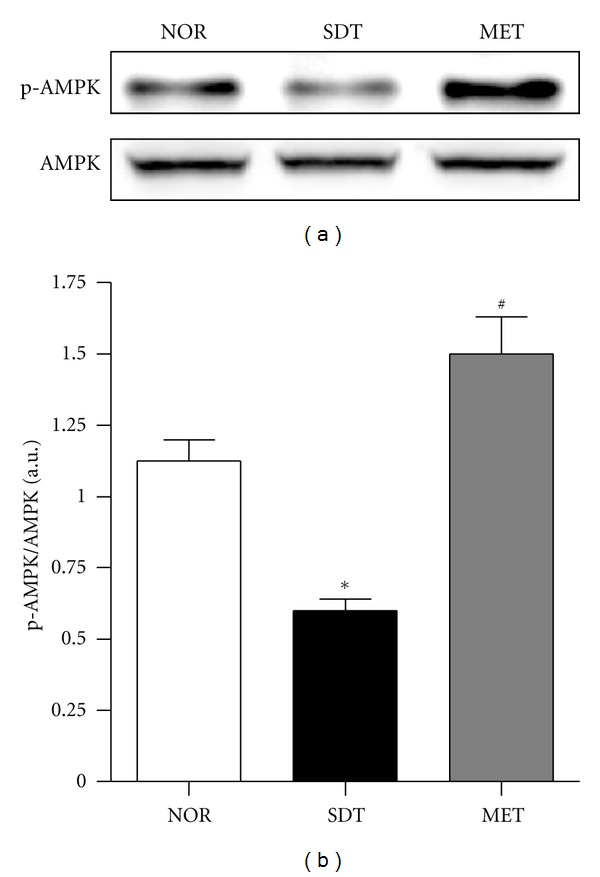
AMPK activation in renal tissues. (a) Representative immunoblots of phospho-AMPK and AMPK in protein extracts from the kidneys of rats of each group. (b) Quantitative analysis of protein expression. All data are expressed as mean ± SE (*n* = 8). *P < 0.01 versus NOR group, ^#^ *P* < 0.01, versus SDT group.

We assessed whether metformin activates AMPK in the kidney using immunoblotting for phosphorylation of AMPK. Activation of AMPK was significantly reduced in the kidney of SDT rats compared with normal rats, and metformin induced AMPK activation in the kidney (Figures 4(a) and 4(b)).

## 4. Discussion

Animal models traditionally employed in the investigation of diabetic nephropathy include STZ-induced diabetic rats, but the general pathophysiology of disease in these models differs from that of NIDDM in man. The SDT rat, a model of nonobese type 2 diabetes, was established in 1997 by Shinohara et al. [18]. Hyperglycemia spontaneously develops in SDT rats, predominantly due to an insulin secretary defect. In addition to marked hyperglycemia, renal complications are a characteristic of SDT rats [20]. Based on the findings of the present study, SDT rat may be an appropriate an animal model for investigation of diabetic nephropathy. This model has many similarities to human NIDDM and the development of diabetes mellitus in SDT rats is accompanied by functional and morphological kidney damage that resembles human diabetic nephropathy [20, 21].

Growing evidence suggests that the overproduction of ROS may be the key initiating event that leads to the long-term development of diabetic complications [22]. However, the specific mechanisms that link hyperglycemia with oxidative stress and diabetic nephropathy are poorly understood. In general, oxidative stress can affect nucleic acids and generate various modified bases in DNA. 8-OHdG is the most abundant and appears to play a crucial role in mutagenesis [23]. Oxidation of guanine to form 8-OHdG acts as a marker of oxidative DNA damage [24]. When DNA is damaged, cells initiate a response, such as DNA repair, cell-cycle delay, or induction of apoptosis [25]. Increased levels of 8-OHdG have been reported in urine [26], mononuclear cells [27] and skeletal muscles [28] of diabetic patients. The levels of 8-OHdG were increased in kidney tissues of STZ-induced diabetic rats [29]. In the present study, we further revealed that levels of 8-OHdG were increased in renal tissues of SDT rats. To localize expression of 8-OHdG in diabetic kidneys, kidney tissues were analyzed by imunohistochemical staining. Although the method for 8-OHdG detection stains nuclear DNA as well as mitochondrial DNA (mtDNA), the staining was localized mainly in the cytoplasm, indicating that this oxidative adduct was present in mtDNA, but not in nuclear DNA. It is accepted that vulnerability to oxidative damage and subsequent mutations is 10–20 times greater for mtDNA than for nuclear DNA [30, 31]. In parallel with the results of renal tissues, the amounts of urinary 8-OHdG excretion were markedly increased in SDT rats. Although urinary 8-OHdG levels are supposed to be markers of the total systemic oxidative stress in vivo [32], the relative contribution of renal 8-OHdG to urinary 8-OHdG should be evaluated in future studies.

The generation of ROS through oxidative stress causes cell death [33]. Apoptosis, which is characterized by chromatin condensation, DNA fragmentation, and the activation of caspases, has been implicated in the pathogenesis of various renal diseases, including diabetic nephropathy [34, 35]. ROS are potent inducers of apoptosis in various cell types including murine and human podocytes [36–38]. Our present study shows that the density of podocytes decreases in SDT rats in association with increased albuminuria. Podocyte apoptosis has been demonstrated to correlate with worsening albuminuria [38]. Furthermore, strong evidence has established a role for intracellular ROS as potent inducers of podocyte apoptosis [39]. The increase in antioxidant enzymes such as heme oxygenase-1 has been shown to reduce podocyte apoptosis under diabetic conditions [40]. Consistent with this interpretation, the results of the present study demonstrate that the increased 8-OHdG concentration in affected renal glomeruli is involved, at least in part, in the injury of podocytes.

In addition, we examined the effect of intervention by metformin treatment on the increases in 8-OHdG levels and podocyte loss in kidney tissues of diabetes. It is of great interest that intervention by metformin treatment inhibited both increases in renal 8-OHdG levels and podocyte loss in kidney of SDT rats. The treatment of SDT rats with metformin slightly decreased blood glucose and HbA1c levels. Hyperglycemia, a key clinical manifestation of diabetes, is supposed to generate ROS through various mechanisms, such as increased formation of advanced glycation end products (AGEs) [41], enhanced polyol pathway [42], increased superoxide release from mitochondria [43], and activation of NAD(P)H oxidase [44]. In our present study, metformin induced only a minor decrease of levels of blood glucose (22%) and HbA1c (23%) in SDT rats. Similarly, metformin failed to reduce blood glucose levels in ZDF rats [45]. Although metformin has a weak glucose-lowering effect in SDT rats, metformin has significant effects on any parameters of renal structure and function without the strong reduction of blood glucose. The level of albuminuria was decreased by 63% in metformin-treated SDT rats. However, Fujii H et al. showed that the treatment of insulin decreased the level of HbA1c by almost 55% [46]. The level of albuminuria was also decreased by 62% in insulin-treated SDT rats. These findings suggest that even in hyperglycemia, it is possible to attenuate podocyte injury by metformin.

Metformin has been found to activate AMPK, a major cellular regulator of lipid and glucose metabolism [13]. Previous studies also have shown that metformin is an inhibitor of complex I of the mitochondrial respiratory chain independent of the AMPK pathway [47, 48] and a potent inhibitor of AGE formation [49]. AMPK pathway acts as a signal for ATP generation, a process coupled to increase in ROS production. AMPK pathway can be activated by intracellular ROS and reduces ROS levels [50, 51]. The ability of AMPK to simultaneously reduce ROS levels and counterbalance the overproduction of ROS is an important mechanism for controlling the redox balance during energy production. In present study, we found that the phosphorylation of AMPK was reduced in the kidney of SDT rats, and metformin could restore its alteration. Therefore, metformin is likely exerting some of its effects via improvement of renal oxidative stress. It could be reasonable to assume that the renoprotective effect of metformin might be at least partly, attributed to its influence on AGEs formation and ROS production, besides its antihyperglycemic effect. These observations suggest a potential clinical use of metformin in the prevention of diabetic nephropathy by inhibition of AGEs and improving the free-radical defense system. Liu et al. have pointed the beneficial antioxidant effects of metformin in STZ-induced DM in rats [52]. 

In conclusion, SDT rat is a useful model for the evaluation of the effects of drugs and the investigation of diabetic nephropathy. We also demonstrated that metformin protects against diabetic nephropathy by restoring the biochemical alterations and modulation of oxidative stress, and hence suggests a potential clinical use of metformin in the prevention of diabetic nephropathy.

## Figures and Tables

**Table 1:** Metabolic and physical parameters.

	NOR	SDT	MET
Body weight (g)	713.9 ± 29.7	375.5 ± 52.4*	430.5 ± 13.4
Blood glucose (mg/dL)	144.1 ± 21.0	419.2 ± 21.2*	359.5 ± 52.3
HbA1c (%)	3.49 ± 0.07	9.13 ± 0.37*	7.82 ± 0.29

NOR: normal rat; SDT: spontaneously diabetic Torii rat; MET: SDT rat treated with metformin (350 mg/kg/day). All data are expressed as mean ± SE (*n* = 8). *P < 0.01 versus NOR group. ^#^ *P* < 0.01 versus SDT group.
